# Dating with a Diagnosis: The Lived Experience of People with Multiple Sclerosis

**DOI:** 10.1007/s11195-021-09698-9

**Published:** 2021-05-26

**Authors:** Kinza Tabassum, Jackie Fox, Sara Fuller, Sinéad M. Hynes

**Affiliations:** Discipline of Occupational Therapy, School of Health Sciences, Áras Moyola, National University of Ireland Galway, Galway, Ireland

**Keywords:** Multiple sclerosis, Dating, Romantic relationships, Diagnosis, Ireland

## Abstract

Multiple Sclerosis (MS) is a neurological condition which usually manifests between the ages of 20–40 years. This is a critical period for developing relationships, particularly romantic relationships. People with MS can experience sexual dysfunction, limb weakness, fatigue, pain, reduced mood and bladder/bowel dysfunction; potentially affecting their ability to participate in many meaningful activities, including those associated with romantic relationships, dating or engaging in sexual intercourse. Dating or starting romantic relationships can be difficult for people with physical disabilities as they can experience stigma, negative societal attitudes and the fear of requiring care from potential partners. Dating experiences of people with progressive conditions like MS have not been explored in detail. The aim of this study was to develop a rich understanding of how living with MS interacts with/influences dating and developing romantic relationships. The study used a descriptive phenomenological design and a purposive sampling strategy. Colaizzi’s descriptive phenomenological method was used to analyze the data (Colaizzi, 1978). Five females and two males, aged 23–51, participated in two online focus groups. Dating with a diagnosis of MS is a highly personal phenomenon, characterized by individual differences in values and experiences. Core to the phenomenon was personal decision-making about disclosure of the diagnosis and ongoing adaptation to the fluctuating nature of the condition with partners in new/developing relationships. The findings will help health professionals working with adults with MS understand this important aspect of their lives.

## Introduction

Multiple sclerosis (MS) is a progressive neurological condition which primarily affects the central nervous system. Common symptoms include fatigue, limb weakness, cognitive deficits and difficulties in speech, vision, bowel, bladder and sexual function [2]. Due to the complex and unpredictable nature of the condition, symptoms often vary in severity and presentation [3]. MS can significantly impact a person’s ability to engage in various meaningful activities, including those related to dating and relationships. Research to date has focused on the impact of MS on marriage or long-term relationships [4–7] However, there is currently a gap in the literature exploring the dating experiences of people with MS.

A recent review of the literature explored the impact a diagnosis of MS has on familial and social relationships [3]. Participants described that with time, the physical and psychological presentations of MS created a need for care which impacted their independence and altered roles and responsibilities in relationships. For example, their family members began to take on a caregiving role [3]. In the context of marriage and long-term romantic relationships, the impact of MS manifested as reduced participation in shared activities and reduced emotional closeness with their partner [3].

The experiences of marriage and long-term relationships for people with MS are diverse and the extent to which the condition impacts relationships varies from person to person. From their research of the impact of MS on interpersonal relationships, Herbert et al. [8] found that most participants felt their relationships became closer as a result of MS or that MS had no significant impact on their relationships. However, for some individuals, the diagnosis and symptoms of MS posed challenges which damaged their relationship or contributed to the ending of their relationship. A study by Landfeldt et al. [9] suggests that a MS diagnosis is associated with a higher risk of divorce among married couples.

A recent Iranian study illustrated that an MS diagnosis can create a fear of separation or rejection from long-term romantic partners [10]. Individuals also described the impact of physical and psychological changes on their relationships such as reduced intimacy with their partners or behavioral changes, for example increased irritability toward their partner. In relation to dating or forming new relationships—participants perceived themselves as less desirable due to their diagnosis and associated this with a reduced possibility of marriage in the future. As a result, in most cases, participants preferred not to disclose their MS to potential partners. A diagnosis of MS also changed their criteria for partner selection. For example it became more important to find someone calm and supportive. MS usually manifests during early adulthood, between the ages of 20–40 years. This is a critical period for the development and maintenance of romantic relationships through dating [8, 10]. Dating can be defined as “the process of finding a romantic partner(s), which might involve many meaningful activities such as: dressing, social activities, online dating, meeting someone for coffee etc.” [11 p.2]. To date, there has been little research exploring the lived experience of people with MS as they engage in dating and its related activities.

Although research exploring the dating experiences of people with MS is sparse, studies involving those with physical disabilities demonstrate how the stigma associated with an illness can impact the formation of romantic relationships–where dating someone with an illness is perceived as stressful or associated with anxiety, stigma-by-association or a burden of care [12]. From the perspectives of individuals with physical disabilities; physical attractiveness, self-worth issues [13] or negative societal attitudes such as being perceived as asexual or helpless, have also posed a barrier to dating [14].

The dating experiences of people with MS may be very different to the experiences of individuals with physical disabilities because many of the condition’s symptoms are invisible. Research shows that invisible symptoms of MS such as pain, fatigue and cognitive problems have a significant impact on social interactions as they create a mismatch between how the individual appears on the outside and how they are feeling internally [15]. These invisible symptoms are often perceived as the most distressing and difficult symptoms of MS as they can result in under-recognition of their condition during social interactions [15, 16].

Research to date has shown that the diagnosis and symptoms of MS can impact family relationships, marriage, and long-term relationships [9, 10]. However, very little is known about the experiences of people with MS as they partake in the occupations associated with dating and form or maintain romantic relationships [10]. This study is being carried out in response to the lack of research around this phenomenon to elicit valuable insights. Obtaining the perspectives of people with MS related to dating can provide key information which may help healthcare professionals address any challenges and improve the quality of engagement and participation in occupations that are associated with dating.

## Methods

### Study Design

This qualitative study used descriptive phenomenological methodology to develop a rich understanding of the impact of MS on romantic relationships and dating. A qualitative research design is considered appropriate when the aim of the research is to explore the subjective or lived experiences of participants in depth [17]. Due to the paucity of existing literature in this area, descriptive phenomenology was chosen as the overarching approach to allow for a rich, in-depth exploration of this poorly understood phenomenon [18]. In descriptive phenomenological studies, care is taken to describe the phenomenon as it appears to participants, without adding details or explanations that are “not given” by them i.e. avoiding interpretation, theoretical viewpoints, making assumptions or holding hypotheses [19]. To ensure this was achieved, the authors identified and questioned their pre-understanding of the dating phenomenon to ensure that the authentic lived experiences of the participants were brought to the fore [20]. They reflected on their own assumptions, values and life experience through reflective journal writing prior to the study and continued to maintain a reflective and questioning attitude throughout, for example, by holding joint data analysis meetings where different understandings of the meaning of participants’ statements could be debated and clarified.

### Participants

A purposive sampling strategy was implemented to identify participants with relevant knowledge or experience to address the research topic. This allowed the authors to capture a rich, thick and in-depth description of the phenomenon. The researchers attended to the context as well as detailed description and interpretation of the phenomenon. Participants were recruited from the support organization MS Ireland through their social media accounts (Facebook and Twitter), website and mailing lists. Participants who were interested in partaking in this study self-recruited by contacting the authors and were eligible to participate if they: (i) were aged 18 or over (ii) had a diagnosis of MS (iii) were able to communicate in English and (iv) had an email address and computer or smartphone access to participate in the online focus group. Participants were excluded if they (i) were co-habiting with a partner or married (ii) had a comorbid diagnosis of a neurological disorder or (iii) were not able to provide informed consent. Nine people expressed an interest in the study and seven consented to participate.

### Procedure

Ethical approval was obtained from the Research Ethics Committee at the National University of Ireland, Galway (REC reference: 19-Dec-16). Two online focus groups were conducted in June and July 2020 using the online video-conferencing software Zoom. After giving written consent, participants were asked to fill out demographic details through an online, secure form prior to the interviews. Each focus group lasted for approximately one hour and was conducted at a time that was convenient to participants. The focus groups were facilitated by two of the authors, with one leading and one taking field notes or providing technical assistance if required.

Focus groups can elicit high quality qualitative data where the information gathered is mainly generated through the discussion between participants [21]. Due to the lockdown in place in Ireland at the time of the study because of the COVID-19 pandemic, online focus groups were considered to be the safest and most appropriate method of data collection. Research shows online focus groups can elicit the same quality of data as in-person focus groups [22]. This method of data collection also allowed recruitment of participants from a larger geographical area and reduced some of the limitations associated with in-person focus groups such as travel, cost and unfamiliar environmental context.

The development of interview questions was guided by Bevan’s method [23] of carrying out phenomenological interviewing. In line with this method, questions were structured to first place the phenomenon in context, moving on to apprehend the phenomenon and finally clarifying the phenomenon [23]. Expert review of the interview guide (see Appendix) and consultation with a patient representative ensured that the questions designed were appropriate for data collection. The co-facilitator made detailed contextual notes throughout the focus groups to record important details which otherwise could not be captured solely through verbatim data, for example, gestures and facial expressions. The authors engaged in reflexive analysis following each interview to make note of initial thoughts and observations. Examples of the interview questions are given in Table 1.

**Table 1:** Development of the descriptive phenomenological interview [23]

Contextualising questions–broad description of the phenomenon	Followed by apprehending the phenomenon more fully—eliciting more detailed descriptions of pertinent events/experiences	Finally clarifying the phenomenon—understanding what the phenomenon is not, or when there are changes to the experience
Can you describe for me what it is like to date with MS?	Can you describe for me how you find dates? What is that experience like?	How have things been different since COVID-19? How has your dating life changed? (if it has?)
Can you describe for me any experiences that would give me a good idea of what it is like?	Can you describe what the early stages of relationships are like with MS, from your experience?	

### Data Analysis

Following verbal and written consent from participants, audio-recorded data from the focus groups was anonymized and transcribed verbatim. The authors used Colaizzi’s [1] descriptive phenomenological method to analyze the data (see Table 2 for example). Memos were used to track emerging thoughts and maintain reflexivity throughout data analysis. Data analysis was completed independently by the authors and emergent findings were discussed during joint data analysis meetings. Using Colaizzi’s [1] seven-step method, the authors independently analyzed the data by (1) reading and becoming familiar with the data (2) identifying significant statements relevant to the phenomenon of dating (3) formulating meanings (4) clustering themes (5) developing a rich description of the phenomenon (6) producing the fundamental structure and (7) completing member checking by returning the rich description back to participants to verify if it was an accurate analysis of their lived experience [18].

**Table 2:** Example of data analysis process using Colaizzi Method.

Data analysis step	Identifying significant statements	Formulating meanings	Clustering themes	Exhaustive description	Fundamental structure
Explanation	*Sections of verbatim transcripts where key points were made*	*Analysing significant statements to identify meanings related to the phenomenon*	*Grouping identified meanings into themes that appear frequently*	*A detailed description of themes related to the phenomenon*	*Summary of the exhaustive description encompassing the essential composition of the phenomenon*
*Example*	*“You can’t relax into something properly until you’ve gotten that bit out of the way. It’s just always in the back of your mind until it comes up–like how is it gonna come up, when is it gonna come up, what’s their reaction gonna be, cause there’s always a possibility that it’s gonna be bad” [P3]*	It is difficult to relax into or feel comfortable in a relationship until the MS diagnosis is disclosed. It is something that is always on the back of one’s mind until it comes up. You wonder how it will come up, when it will come up, or what the other person’s reaction will be. There is a fear that their reaction could be bad	Fear of rejection associated with disclosing that you have MS	Figuring out exactly when it is appropriate to disclose the diagnosis in a developing romantic relationship can be difficult and requires careful thought and consideration of the circumstances under which it should be disclosed	Deciding when and whether to disclose the diagnosis in a developing romantic relationship is personal and requires careful thought and consideration about the circumstances under which it should be disclosed

### Trustworthiness

Credibility was ensured through investigator triangulation and member checking (occurring at two stages during the analysis process). Dependability and confirmability were ensured through detailed description of the research steps in an audit trail. The authors ensured that reflexive bracketing was done consistently throughout the research process in order to be self-aware of their own preconceived ideas, beliefs or assumptions which they may bring to the research. Reflexive bracketing is a core element of descriptive phenomenology which allows the researcher to separate their own subjective views from those of the participants, to allow for an accurate account of the essence of the lived experience of the phenomenon, thus enhancing validity of the findings [24]. Finally, the COREQ (Consolidated criteria for reporting qualitative research) [25] checklist was also used to aid with reporting of the research and enhance rigor and trustworthiness.

## Results

Five females and two males participated in the study. Five participants took part in the first focus group and two participants took part in the second. Participants were allocated to groups based on their availability. Participants were aged between 23–51 years. Six participants had a diagnosis of relapsing–remitting multiple sclerosis and one participant had a diagnosis of primary progressive multiple sclerosis. Years since MS diagnosis ranged from 4–16 years.

The output of a study using Colaizzi’s descriptive phenomenological method is a “fundamental structure” which is a synopsis of the phenomenon–closely linked to the participants’ description, without the overlay of researcher interpretation [1, 26]. This structure is organized under four themes and was confirmed and added to by the participants during the member checking process:

1. Disclosing the MS diagnosis is a personal choice
2. There is a negative perception associated with having MS that you firstly need to address yourself
3. MS can impact dating and dating activities
4. Online dating can be intense, but COVID-19 has been a leveler

### Disclosing the MS Diagnosis is a Personal Choice

Dating with a diagnosis of MS is a highly individual phenomenon, characterized by differences in values and experiences. One of the key experiences is the disclosure of the MS diagnosis to a potential partner. Deciding when and whether to disclose the diagnosis in a developing romantic relationship is personal and requires careful consideration about the circumstances under which it should be disclosed.

*“You can’t relax into something properly until you’ve gotten that bit out of the way. It’s just always in the back of your mind until it comes up – like how is it gonna come up? When is it gonna come up? What’s their reaction gonna be? Because there’s always a possibility that it’s gonna be bad” (P3)”*.

Different strategies are used to disclose the MS diagnosis which are unique to the individual and their experiences. Some people choose to disclose their diagnosis clearly at the same time as they disclose another important aspect of their life, for example, a child from a previous relationship. Others prefer to disclose their MS indirectly or “naturally” through their social media or hide their surname until they feel comfortable with the person finding out more about their lives.

*“When I feel more comfortable I go: “Oh ok my second name is ___” and they usually look it up, and it’s like this girl… like MS is like nothing. She’s having a great time like you know? (laughs) They come back to the [person] that they got to know previously“ (P1).*

The decision of when and how to disclose an MS diagnosis is also based on perceptions of the potential partner. Some prefer to disclose the diagnosis straight away during the initial stages of a romantic relationship as they believe there is no point in developing a relationship with someone if the MS is not something that the potential partner can deal with. To give time for this decision-making, other people may prefer to wait until later in the relationship before they introduce their MS. This allows the individual to get to know their potential partner, become more comfortable with them and judge whether they possess the characteristics that they would want in a future partner. Sometimes, MS comes up naturally in a conversation and is introduced in this way.

For some people, early disclosure is used as a way of avoiding individuals who seek out relationships with people with disabilities—who view the person with the disability as a “tragic victim” or “damaged” and want to “rescue” that person or want them to be dependent. There is a fear for some that revealing the diagnosis before meeting someone may give rise to preconceived ideas–that the person with MS will have a physical disability and will *“come in with a stick or a chair (P2)”.* It was agreed that MS does not reduce your value as a person or mean that you will settle for less in a relationship. Everyone has something in life that they struggle with, which they bring to the table when dating–and MS is no different; “*the unpredictability of MS makes it[dating] far more interesting. Perfect is boring*” (P5).

Some people believe that if they do not view their MS as an issue themselves, there is no reason why anyone else should. The MS did not change standards or the type of people one is interested in. Embracing a hardship such as MS and handling the condition with a positive mindset can tell a lot about a person’s character early in a romantic relationship–it shows the partner how the person deals with negative situations or hardships in life early in a romantic relationship instead of months or years down the road.

“*If you’re going out with someone and don’t mention your MS like it’s a problem, mention it like it’s something interesting you beat everyday–that’s what I do*.” (P5)

There can be a fear of rejection associated with the disclosure of the MS diagnosis. Until the diagnosis has been disclosed, it can be difficult to feel comfortable or secure in a new relationship as there is uncertainty around what the partner’s reaction will be–sometimes it may go well and other times it may not. In some circumstances the disclosure of an MS diagnosis contributes to the ending of relationships. As a result of this some people fear that having MS will reduce the likelihood of someone wanting to date them. The emotional impact of rejection due to MS can be significant especially if the person was emotionally invested in the relationship;*”you think you’re getting on well with somebody and then you drop the MS and they’re just gone… which is quite soul destroying” (P4).*

Those who have experienced rejection due the MS diagnosis sometimes decide to hide this fact–covering up the symptoms as something else, or avoiding disclosure for as long as possible: *“It complicates things in my own mind and also I believe it will be in other people’s as well, so I just don’t tell anybody anything (P4)”*. This is done to be judged without MS, rather than being intentionally untruthful.

*“I wanna be judged as a person–not as a person with MS, which to me and to other people does have some negative connotations with it, so how do I tell people?(P4)”.*

However, it is acknowledged that not sharing the MS diagnosis until much later on in a relationship can lead to other problems such as feelings of betrayal and mistrust. A potential way of avoiding such complications for some is to be open about their MS diagnosis on social media.

“…*that is why I give them Instagram or something like that cause then … my Instagram is everything from paragraph-long MS posts to drunken pictures from nights out. You can see the whole person. But I almost do that to avoid [a negative reaction]. If they stop talking to me, I don’t have to necessarily know if it was my MS or not because people ghost you at the best of times, even if you haven’t told them that you have MS*” (P3).

Finally, it is common for people to date someone within their friendship groups, which means that it is likely that their partner will already know about their MS diagnosis. During the initial period after receiving MS diagnosis, some people abstain from dating, not wanting to introduce strangers into their lives at that point in time.

### There is a Negative Perception Associated with having MS that you Firstly Need to Address Yourself

Reactions to receiving the MS diagnosis can be mixed. Sometimes it is difficult to deal with the emotional impact of the diagnosis because it can make people feel vulnerable, frightened and uncertain about how it will impact their life–including their romantic relationships. Having the right information at the time of the diagnosis is essential to avoid *“a full-on freak out” (P6).* Others accept their diagnosis more readily as it is a relief to know why they’re experiencing certain symptoms. Being in a relationship while going through the process of diagnosis can be taxing because of the extra burden of dealing with the partner’s emotional response or reassuring them.

*“I was quite pleased to be single because then I didn’t have a partner that… would almost need reassuring I’d rather have gone through it myself got it sort of sorted in my own head first… rather than becoming… sort of wrapped up with a partner’s attitude and their approach and how it affects the relationship (P7)”.*

For this reason, there can be relief (for some) not to be in a relationship at the time of receiving the diagnosis. This allows the person to come to terms with their MS first in their own head without the emotional labor of dealing with someone else.

People’s perceptions of MS are often tied up in images of disability. These images or false perceptions arise from a complex mix of outdated or previous generational experiences of MS, worst-case scenarios portrayed by MS fundraisers and media portrayal of people with MS. There is also a perception that the person with MS will “eventually die”, therefore one should be cautious about becoming invested in or romantically involved with them.

These false perceptions often must be addressed during dating. Constantly educating people about MS can, however, become emotionally draining and exhausting and it can be a challenge for participants to manage the “*emotional labor of dealing with someone else’s fallout*” (P6) when learning about MS. This is viewed as even more challenging if the type of MS is not commonly known, for example primary progressive MS, or if the diagnosis is very recent, as the person themselves is still in the process of learning about their MS and how it affects them. Therefore, some people will only go into detail about their condition if they are emotionally invested in that person.

“*I would wait a while just because it is emotionally tiring trying to deal with somebody else’s response to it and then kind of reassuring them and it’s like, do I really want to be spending my time reassuring somebody? (It) is like almost twice as much energy is invested into reassuring somebody. I don’t feel there is a big problem but the somebody else kind of requires me to kind of look after them to make sure they’re okay with it*” (P7)

The information available to the public about MS is also viewed as unhelpful in this regard, as it means that you have to explain which symptoms are relevant to you and which are not. The information available about MS can be alarming and can leave potential partners who research the condition misinformed. People are reluctant to point others to any resources or encourage others to do their independent research–the person with MS themselves will always remain the best source of information about their MS.

### MS can Impact Dating and Dating Activities

MS symptoms such as trigeminal neuralgia and hypersensitivity can impact the physical intimacy between a couple. Hypersensitivity in areas of the body can make physical affection uncomfortable for the person with MS. This can have an emotional impact; *“it feels awful …to shudder at the touch of your partner, tell them to stay away from you and*…*it isn’t nice”* (P2).

The symptoms of MS may complicate the practical aspects of dating. Dating activities may be limited or restricted due to pain or fatigue–these may range from simple activities such as going for walks to more complex activities such as climbing. As a result, dating activities have to be chosen carefully and planned in advance to consider the practicalities of the date such as parking, suitability of the building or place, length of the activity and strategies which may be used to adapt such as taking breaks during a walk, drinking coffee to counteract the fatigue. Pre-date planning about these adjustments can also use up a lot of energy.

Adapting to the unpredictable nature of the condition may be challenging for couples as they adjust to the day-to-day uncertainties. This can be frustrating and is thought to have a greater impact on the partner, as the person with MS may be more used to the fluctuating nature of their condition. It takes a certain “*type*” of partner to deal with this—someone who is patient, understanding and willing to learn how to adapt to the uncertainty of the condition:

*“I think there are certain types of people that just wouldn’t be able to deal with the day to day… you just have to be patient, you have to be understanding (P2)”.*

Clear communication with your partner and developing strategies to facilitate understanding of the condition is important. This could include being honest and straightforward about last-minute changes to plans (otherwise it could create a misunderstanding that there is something wrong with the partner or the relationship). Involving the partner with the management of the condition such as bringing them to MRI appointments or blood tests is also helpful as it allows the partner to better understand how the MS may or not impact their partner.

### Online Dating can be Intense, but COVID-19 has been a Leveler

Online dating apps or websites are frequently used amongst people with MS. Online dating can provide an opportunity to meet more people, particularly for those living in rural areas. The experiences of online dating are varied–sometimes it is easier or more efficient, but it can still pose challenges.

Online dating is preferred by people who do not go out very often and therefore don’t have many opportunities to meet potential partners in person. Online dating may be viewed as sequential–people transition from getting to know each other to developing a romantic relationship and then meeting for in-person dates. Online communication like voice messages can help to get a clear sense of the person and allows people to get to know each other without judgement. Some people find it easier to be expressive online compared to in-person. Online dating also provides the opportunity to meet a larger variety of people for dating.

For some, online dating is more intense or emotionally difficult as it requires strength to put themselves out there. Online dating can be more work compared to in-person dating as the process may take more time and could be construed as similar to business networking. Finally, some people believe that online dating is not realistic. This is because people may portray a false online persona which may not match what they are like in real life.

*“… you imagine them to be more than they are–it’s like reading a book, reading a book is always better than watching a film because you imagine it the way you want and somehow you brain decides that that is what this person looks like this is how they are and you do it for yourself so you always think of very positive things but when you meet them in person it’s not the same (P4)”.*

Furthermore, some aspects which may be obvious in real life may not be easy to figure out through an online dating profile. Therefore, for some people, meeting in real life is more important to understand the character and personality of the potential partner.

Online dating is done in phases and it may suit a certain stage of life, for example if you move to a new location, or are older. Some people believe that it is easier to date with MS when older, as they come to an acceptance of their condition, become more grounded in themselves and realize that they do not need a relationship in order to feel fulfilled. A shift in perspective occurs–the MS is viewed as a major issue when younger, however with age there is a realization that the MS is only one part of them as a whole person. Finally, dating can be easier when older as people gain life experience and as a result, become more tolerant or accepting of other people’s “perceived flaws”. This acceptance makes it easier to share the diagnosis with people.

The COVID-19 pandemic has not had a significant impact on dating experiences of participants, although it may have given people a push to start dating online. Lockdown limits dating options and makes it more difficult to meet people in real life. Long distance relationships are possible during COVID-19 due to online communication. Online dating during COVID-19 has been positive– COVID-19 is considered “*to be a great leveler*” as everyone is now doing the same thing for dates. There is more choice, as more people are engaging with dating apps and websites during COVID-19—though many people who prefer in-person dates over online dating may have lost interest in dating during COVID-19.

## Discussion

Findings from this study show that, overall, dating is a phenomenon experienced differently by people, depending on their past experiences and outlook. However, decision making around the disclosure of the diagnosis is central to the lived experience of dating. People choose when to disclose their diagnosis and how they disclose their diagnosis based on personal values, beliefs and most importantly, previous dating experiences. Some people prefer to disclose the diagnosis in the early stages while others may prefer not to disclose their diagnosis at all due to negative reactions from partners experienced in the past. Research has found [27] that a significant number of people with MS will go out of their way to keep their MS diagnosis hidden from others. These findings are reflective of the online dating experience of people with disabilities reported elsewhere [28]. For people with physical disabilities, engaging in online dating forces them to confront their disability as part of their identity [28]. For people with chronic health conditions or disability, it is evident that there is a laborious and intense process involved in (online) dating that involves understanding and navigating group norms [29].

Disclosure of MS diagnosis is often considered to be “high risk” and can have negative implications, particularly in the context of employment [30]. Participants in the current study also spoke about the risks involved in disclosing their diagnosis early on when building relationships. Because MS is often seen as an “invisible” disability [31] there is a need to disclose at some point, unlike disabilities which can be seen. There can be an expectation that people disclose more severe and “visible” disabilities to future partners more so than those considered to be “invisible” [29]. Interestingly, here we found that some participants used the process of diagnosis disclosure as a way of filtering out people that they would not want to date or for whom MS would not be something they would be willing to deal with. This method of proactive disclosure has also been reported as a way of “*weeding out those who aren’t mentally or emotionally equipped to date someone with a disability*” [29, p 8]. Also, in the current study, people expressed that having MS did not mean that they had to now change their standards or settle for less, which is in contrast to a similar Iranian study [10].

Grytten and Maseide [32] consider one’s identity and sense of self to be central to the decision to disclose a diagnosis. In the current study, it is clear that they thought it was important for them to be comfortable with their own diagnosis before they went through the process of disclosure with others. People with MS incorporate their diagnosis into their identity, but it takes time [33]. This process can have a positive impact on mood and happens more easily when the diagnosis itself is not stigmatized [33], which was not the experience of the participants in the current study. Participants spoke about how it can be exhausting to go through the process multiple times with different people. Not disclosing can, however, also have significant negative impacts on people. Emotional and psychological strain of concealing this diagnosis can often be challenging [34, 35] and for some people it is less burdensome to disclose than to conceal their diagnosis. However, a minority experience amongst people in this study was continuing to hide their MS out of fear of being judged or treated differently.

The process of disclosure can be associated with ethical challenges where some people report a moral dilemma in relation to being truthful, or otherwise, with regards to their health status [30]. The (ethical or otherwise) decision to disclose is often balanced with the person’s fears that are intertwined with the disclosure. A global study of 1075 people with relapsing remitting MS found that participants had significant concerns that their diagnosis would make them less sexually attractive and led them to fear that their partners would leave them [27] and so the experiences reported from participants in the current study are not isolated.

As seen in the current study, the symptoms of MS, together with the unpredictable nature of the condition may interfere with dating activities and physical intimacy and push the person to disclose their condition earlier. The practical aspects of dating are also complicated by the symptoms of MS such as sensory symptoms, pain and fatigue. Therefore, dating activities often require careful planning or adaptation due to the unpredictable nature of MS. The impact of the fluctuating nature of MS on the partner was reflected on, as dealing with the day-to-day uncertainties of the condition requires patience, persistence and understanding. The importance of healthy communication was emphasized to facilitate understanding of the condition and develop strategies to overcome such obstacles. The impact of physical symptoms on relationships is commonly reported [10] and in the current study the understanding between the couple was emphasized as a strategy to facilitate coping with the day-to-day uncertainties. This is especially important considering previous research indicating that people with MS have been found to bring considerable psychological burden into their relationships [36].

## Limitations

Some limitations of this study may impact on the transferability of results. The number of men participating in this study was low. It is known that there is a gender difference in the experiences of men and women with MS during dating and in sexual relationships [5, 6]. Having mixed-gender focus groups may have (i) made participants feel less comfortable sharing common experiences and/or (ii) resulted in lack of data saturation, where not enough men or women’s views were gathered. However, there is evidence that well-conducted, mixed-gender focus groups, even on topics relating to relationships and intimacy, can ensure that both perspectives are considered [37]. Because two potential participants were unable to attend the second group, the focus groups were unbalanced in numbers of participants, with five in the first and only two in the second. It is known that people with a diagnosis of MS may have symptoms on a continuum of severity from mild to very disabling [3]. The experiences of the participants in this study may not represent the experience of other people with MS. The impact of MS on sexual relationships was not discussed by participants in this study and it is not clear whether this was because it was not perceived as an issue or if participants just did not feel comfortable disclosing intimate details in a “mixed” group (or group in general). This is also an area that the majority of healthcare professionals do not proactively discuss with their clients [38, 39] and is underreported and underassessed [40, 41], so participants may not have seen it as an area that was open for discussion.

## Conclusion

In conclusion, this study provides an understanding of the impact of MS, a chronic, progressive condition, on dating and romantic relationships. The phenomenon of dating with MS is shaped by differences in values, beliefs and experiences. The dilemma of deciding when or how to disclose the diagnosis is omnipresent throughout the initial stages of a developing romantic relationship. Disclosing the diagnosis in the early stages allows people to ensure that the MS is something their partner is willing to accept or deal with. However, there is also a fear that disclosing the diagnosis this early may lead to rejection or give rise to preconceived ideas about the person with MS. Therefore, some people prefer to wait until they have developed a deeper bond with their partner or have figured out whether they possess the characteristics they want in a romantic partner.

From this research, it is clear that MS has significant impacts on dating and can make the process of dating and searching for romantic partners more difficult. Further research is required to further explore the phenomenon of dating with MS with a broader population and understand the potential role of healthcare workers in this area.

## Data Availability

Qualitative data (interview transcripts) are not available, as this was not approved by the research ethics committee.

## Focus Group Topic Guide

### Introduction

1. Introduction to project.
2. Introduction to what will happen–no more than 90 min, audio-recording (audio-recorder not yet turned on), what will happen to the audio-recording.
3. Introduction to moderator and co-moderator and their roles.
4. Any questions.
5. Ask for consent again before starting to record

## Turn on the Audio-Recorder Here

### Introductions

(Tell people that no names will be written down–they are just to help us feel comfortable talking with each other today)

- Give names and one thing you are happy to share about where you’re speaking from today. Moderator and co-moderator to go first.

### Just Before We Start–a Bit of Guidance Around the Group

- It is important that you know that we are looking for a range of experiences on this topic, so please be honest.
- You can disagree with someone’s comments or share a different opinion. But please be respectful of other people’s experiences.
- Confidentiality–what is spoken about here is not shared.
- And please–only one person speak at a time. If you’re finding it difficult to come into the conversation–please use raise hand or text in the chat function to let me know that you’d like to come in.
- Has anyone got any other guidelines or things we should agree before we begin–to help you feel more comfortable to speak?

## Start with Contextualizin Questions

We are starting broad-

- Can you describe for me what it is like to date with MS? Can you describe for me any experiences that would give me a good idea of what it is like?
- Can you describe any experiences you’ve had with dating that you’re happy to share?

## Summarize Main Points Before Moving onto Another Question

Move into apprehending the phenomenon (remaining descriptive “describe for me”).

- Can you describe for me how you find dates? What is that experience like?
- How do you judge when to tell someone about your diagnosis?
- Can you describe what the early stages of relationships are like with MS, from your experience?

### Prompts

- (If someone describes a date) Can you describe what you felt or thought at that time? Can you describe what you did then/later?
- You say you felt—Can you describe for me what that was like? How it affected you?

## Introduce New Topic Here

- Can you describe your experience of using dating apps or online dating?
- Do you think having MS impacts on that experience?
- Online vs “in-person” dating–describe for me what that is like for you. What is your experience?
- Can you describe what impact COVID-19 has had on your dating and relationships?
  Move into clarifying the phenomenon (imaginative variation) This is elicited from the interview. But sample questions could be: 
  How have things been different since COVID-19? How has your dating life changed? (if it has?).
- This may already be answered from above question.

### Prompts

- (If someone says a date was unsuccessful) What would a successful or enjoyable date or relationship look like for you?
- (if someone says they have friends without MS who date) is there a difference in the experience of those dating without a diagnosis of MS?
- Is there anything else you wish to mention or talk about in relation to this topic?

## Concluding

- Member checking–so, before we leave, the main ideas that came up today were.
  Last question to everyone–what is the most important thing you’d like to say about what dating is like as someone with a diagnosis of MS?
- Tell people that we will get back to them with a summary of results.
- Thank people for their time.
